# Pollen morphology of the African *Sclerosperma* (Arecaceae)

**DOI:** 10.1080/00173134.2018.1519033

**Published:** 2018-11-20

**Authors:** Friðgeir Grímsson, Johan L.C.H. van Valkenburg, Jan J. Wieringa, Alexandros Xafis, Bonnie F. Jacobs, Reinhard Zetter

**Affiliations:** 1 Department of Palaeontology, University of Vienna, Vienna, Austria; 2 Naturalis Biodiversity Center, National Herbarium of The Netherlands, Leiden, The Netherlands; 3 Roy M. Huffington Department of Earth Sciences, Southern Methodist University, Dallas, TX, USA

**Keywords:** light microscopy, palms, scanning electron microscopy, *Sclerosperma mannii*, *Sclerosperma profizianum*, *Sclerosperma walkeri*, swamp element, tropics

## Abstract

Three currently accepted *Sclerosperma* species appear to produce four different pollen morphologies. *Sclerosperma mannii* and *S. walkeri* pollen share the same distinct reticulate sculpture, but *S. profizianum* produces three different pollen types (microreticulate, fossulate, and perforate). The pollen morphology suggests that *S. mannii* and *S. walkeri* are sister taxa of the same intrageneric lineage. The pollen diversity observed in *S. profizianum* suggests (a) this taxon is unique regarding its pollen diversity despite being a non-heterostylous plant or (b) that circumscription of *S*. *profizianum* as a species may well be in the need of redefinition.

*Sclerosperma* G. Mann et H. Wendl. is a palm genus of only three species, *S. mannii* H. Wendl., *S*. *profizianum* Valk. et Sunderl. and *S. walkeri* A. Chev. (Arecoideae, Sclerospermeae; Dransfield et al. 2008), restricted to tropical central Africa, primarily in swampy habitats (Table I; Van Valkenburg et al. 2008). The first account of *Sclerosperma* pollen by Erdtman and Sing (1957) documented its unique morphology within the palm family (triangular, triporate, reticulate), features often discussed by M.M. Harley in the years 1991 to 2008 through her extensive work with colleagues on the pollen morphology of Arecaceae (Harley & Hall 1991; Harley 1996, 1999, 2004; Harley & Baker 2001; Harley & Dransfield 2003; Dransfield et al. 2008). Despite the number of publications containing pollen descriptions and micrographs of the extant genus, *Sclerosperma*, a detailed characterisation of the pollen morphology of the three species was needed. Also, the taxonomy of this genus was only recently revised (Van Valkenburg et al. 2008), and showed that previously published pollen material often originated in misidentified specimens.

**Table I.** African *Sclerosperma* species and their distribution.

Taxon	Occurence in Africa	Pollen samples used for this study
*Sclerosperma mannii* H.Wendl.	Liberia, Nigeria, Cameroon, Equatorial Guinea, Gabon, Angola, DR Congo	van der Burgt, 1958 (K)
*Sclerosperma profizianum* Valk. et Sunderl.	Ghana, Gabon, R Congo, DR Congo, Angola	Gillet, 279a (WAG; Holotype); Grobbelaar, s.n. (K); Hall & Enti, GC36 150 (K); Profizi, 841 (K)
*Sclerosperma walkeri* A.Chev.	Gabon, DR Congo	Leonard, 1614 (BR)

Note: Data extracted from Van Valkenburg et al. (2008) and Bourobou Bourobou et al. (2016).

Here we describe and illustrate pollen from each of the three currently accepted *Sclerosperma* species, compare their pollen, and highlight the diagnostic features that can be used to distinguish them from each other.

## Material and methods

Flowers of *Sclerosperma* (see Table II) from the herbaria of the Botanic Garden Meise (BR), the Royal Botanic Gardens, Kew (K), and Naturalis (WAG; the National Herbarium of the Netherlands) were prepared following the protocol of Grímsson et al. (2017, 2018). Scanning electron microscopy (SEM) stubs with *Sclerosperma* pollen produced for this study are stored in the collection of the Department of Palaeontology, University of Vienna, Austria, under the accession numbers IPUW 7513/217 to IPUW 7513/222.

**Table II.** Herbarium material used for this study.

Taxon	Collector	Coll. No.	Country	Herbarium
*Sclerosperma mannii* H.Wendl.	X.M. van der Burgt	1958	Cameroon	K
*Sclerosperma profizianum* Valk. et Sunderl.	J. Gillet	279	DR Congo	WAG
*Sclerosperma profizianum* Valk. et Sunderl.	N. Grobbelaar	s.n.	Angola	K
*Sclerosperma profizianum* Valk. et Sunderl.	J.B. Hall & A.A. Enti	GC 36150	Ghana	K
*Sclerosperma profizianum* Valk. et Sunderl.	J.P. Profizi	841	R Congo	K
*Sclerosperma walkeri* A.Chev.	J.J.G. Leonard	1614	DR Congo	BR

Note: Species affiliation according to Van Valkenburg et al. (2008)

## Descriptive palynology

The pollen terminology follows Punt et al. (2007; light microscopy [LM]) and Halbritter et al. (2018; SEM). The classification and author names of extant species follow WCSP (2018). Classification above genus level follows Dransfield et al. (2008) and APG IV (2016). Herbarium materials were assigned to extant species according to Van Valkenburg et al. (2008). Pollen grains of each taxon are described individually. Pollen grains of the three *Sclerosperma* species are also compared with each other in Table III.

**Table III.** Pollen morphology of *Sclerosperma.*

	*S. mannii*	*S. profizianum* (Type A)	*S. profizianum* (Type B)	*S. profizianum* (Type C)	*S. walkeri*
Outline polar view	Straight-triangular to slightly concave-triangular	straight-triangular to slightly concave-triangular	straight-triangular to slightly concave-triangular	straight-triangular to slightly **convex**-triangular	straight-triangular to slightly concave-triangular
Outline equatorial view	Bean-shaped	Bean-shaped	Bean-shaped	Bean-shaped	Bean-shaped
Equatorial diameter (µm in LM)	32–38	35–40	32–38	37–42	35–40
Equatorial diameter (µm in SEM)	27–34	29–35	30–34	31–39	30–35
Polar axis (µm in LM)	9–15	10–14	10–13	10–16	**15–19**
Aperture type	Triporate	Triporate	Triporate	Triporate	Triporate
Aperture position	Sub-apically, distal polar face	Sub-apically, distal polar face	Sub-apically, distal polar face	Sub-apically, distal polar face	Sub-apically, distal polar face
Aperture outline	Elliptic	Elliptic	**Circular** to elliptic	**Circular** to elliptic	Elliptic
Aperture diameter (µm in SEM)	4.5–6.0	**5.0–8.5**	4.0–5.5	4.0–6.5	**5.0–8.0**
Exine thickness (µm in LM)	1.7–2.5	1.7–2.5	1.7–2.5	1.7–2.5	1.7–2.5
Pollen wall (SEM)	Semitectate	Semitectate	**Tectate**	**Tectate**	Semitectate
Sculpture (LM)	Reticulate	Reticulate	**Rugulate**	**Scabrate**	Reticulate
Sculpture (SEM)	**Reticulate** to perforate	**Microreticulate** to perforate	**Fossulate, rugulate**/**verrucate**, perforate	**Perforate, rugulate**/**verrucate** and fossulate	**Reticulate** to perforate
Sculpture distal face (SEM)	**Reticulate** with broad muri and elliptic to triangular to polygonal lumina, **0–6 nanogemmae free-standing columellae per lumina**	**Microreticulate** with broad muri and elliptic to triangular to polygonal lumina	**Fossulate** with tiny circular to slit-like perforations aligned within the fossulae, **sinuous fossulae outlining irregular shaped rugulae/verrucae**	**Perforate**, perforations elliptic to slit-like, perforations often aligned in sinuous rows, **rows of perforations outlining irregular shaped rugulae/verrucae**	**Reticulate** with broad muri and elliptic to triangular to polygonal lumina, **0–6 nanogemmae free-standing columellae per lumina**
Number of lumina/perforations at central distal face (SEM)	**18–25** per 100 µm^2^	**30–35** per 100 µm^2^	Not applicable	**45–55** per 100 µm^2^	**16–25** per 100 µm^2^
Sculpture proximal face (SEM)	**Reticulate** central polar area and mesoporium with **elliptic to triangular to polygonal lumina, 0–6 nanogemmae free-standing columellae per lumina**; becoming **microreticulate** to perforate towards apices	**Microreticulate** central polar area and mesoporium with **elliptic to circular or slit-like** lumina; becoming **nanoreticulate** to perforate towards apices	**Fossulate** central polar area and mesoporium with **tiny circular to slit-like perforations aligned within the fossulae, sinuous fossulae outlining irregular shaped rugulae/verrucae**; becoming **microrugulate to nanorugulate/verrucate** and perforate towards apices	**Perforate** and fossulate central polar area and mesoporium, perforations elliptic to slit-like, **perforations often aligned in sinuous rows, rows of perforations and fossulae outlining irregular shaped rugulae/verrucae**; becoming **microrugulate to nanorugulate/verrucate** and perforate towards apices	**Reticulate** central polar area and mesoporium with **elliptic to triangular to polygonal lumina, 0–6 nanogemmae free-standing columellae per lumina**; becoming **microreticulate** to perforate towards apices
Opercula (SEM)	Nanoverrucate to granulate sublayer and **microreticulate** supra-layer	Nanoverrucate to granulate sublayer and **reticulate** supra-layer	Nanoverrucate to granulate sublayer and **perforate** supra-layer	Nanoverrucate to granulate sublayer and **perforate** supra-layer	Nanoverrucate to granulate sublayer and **microreticulate** supra-layer

Note: All measurements include only those from this study and are given in micrometres. Most diagnostic features appear in bold font.

### 

#### Note regarding the following descriptions

According to Halbritter et al. (2018) an ulcus (pl. ulci) is a more or less circular aperture situated distally on the pollen. Ulci are confined to gymnosperms, magnoliid and monocot angiosperm taxa. Also, according to Halbritter et al. (2018) a porus (pl. pori) is a more or less circular aperture located at the equator or regularly spread over the pollen grain. Pori are confined to dicot angiosperm taxa. Still, in all the literature regarding *Sclerosperma* by M.M. Harley from the years 1991 to 2008 (Harley & Hall 1991; Harley 1996, 1999, 2004; Harley & Baker 2001; Harley & Dransfield 2003; Dransfield et al. 2008) the pollen of this genus was described as porate. In order to avoid confusion the apertures of *Sclerosperma* are here also termed pori and the pollen is regarded as triporate and not triulcerate despite the distal position of the apertures.

*Family Arecaceae Bercht*. *et J. Presl*

*Genus* Sclerosperma *G. Mann* *et H. Wendl.*

**Figure 1.**
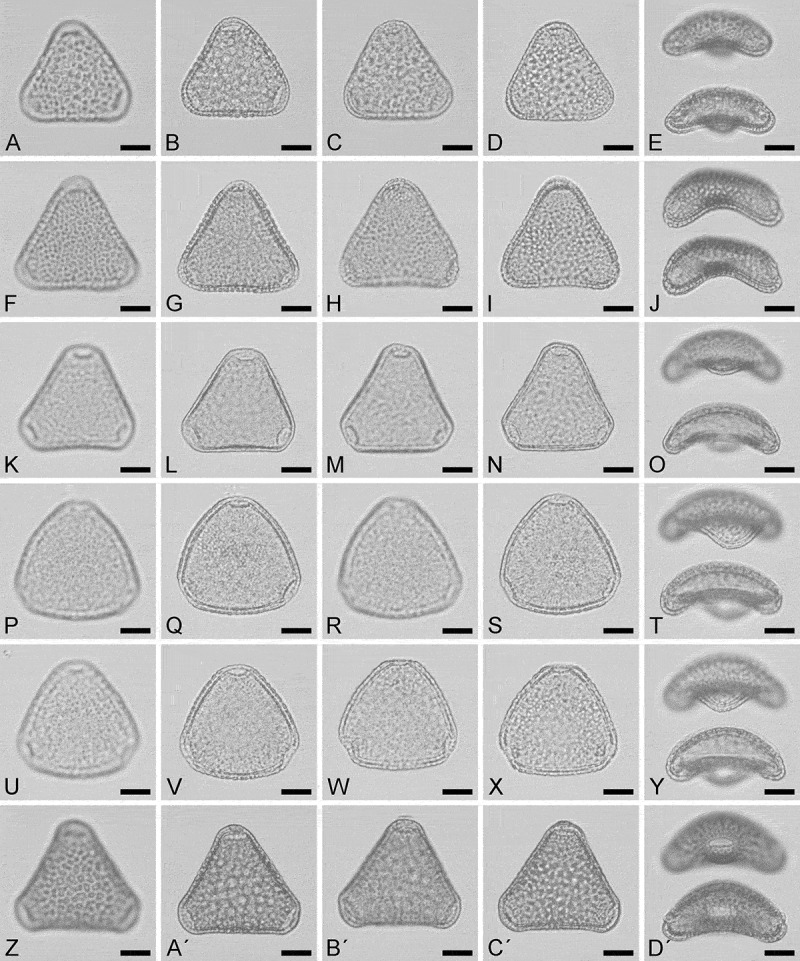
LM micrographs of all *Sclerosperma* pollen types. **A–E.** *Sclerosperma mannii* (from Cameroon, coll. van der Burgt, 1958 [K]), same grain in polar and equatorial view. **F–J.** *Sclerosperma* *profizianum* Type A (from DR Congo, coll. Gillet, 279a [WAG]), same grain in polar and equatorial view. **K–O.** *Sclerosperma profizianum*, Type B (from Angola, coll. Grobbelaar, s.n. [K]), same grain in polar and equatorial view. **P–T.** *Sclerosperma profizianum*, Type C (from Ghana, coll. Hall & Enti, GC 36150 [K]), same grain in polar and equatorial view. **U–Y.** *Sclerosperma* *profizianum*, Type C (from R Congo, coll. Profizi, 841 [K]), same grain in polar and equatorial view. **Z–D′.** *Sclerosperma walkeri* (from DR Congo, coll. Leonard, 1614 [BR]), same grain in polar and equatorial view. **A, C, F, H, K, M, P, R, U, W, Z, B′.** Polar view, high focus. **B, D, G, I, L, N, Q, S, V, X, A′, C′.** Polar view, optical cross section. **E, J, O, T, Y, D′.** Equatorial view, upper in high focus and lower in optical cross-section. Scale bars – 10 µm.

**Figure 2.**
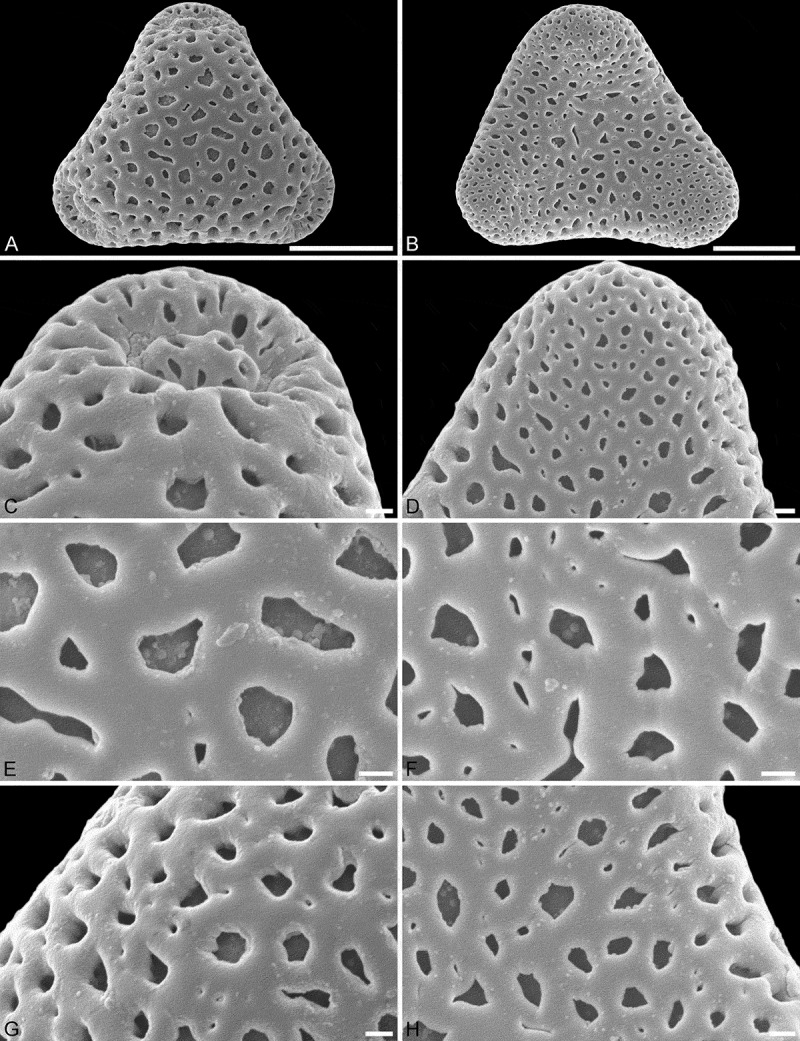
SEM micrographs of *Sclerosperma mannii* (from Cameroon, coll. van der Burgt, 1958 [K]). **A, C, E, G.** Pollen in polar view, distal side. **B, D, F, H.** Pollen in polar view, proximal side. **C, D.** Close-ups of apices (aperture on distal side). **E, F.** Close-ups of central polar areas (reticulum narrower on proximal side). **G, H.** Close-ups of interapertural areas. Scale bars – 10 µm (A–B), 1 µm (C–H).

*Species* Sclerosperma mannii *H. Wendl. (van der Burgt*, *1958 [K])*

(Figures 1A–E, 2; Table III)

#### Description

Pollen, monad, heteropolar, polar axis/equatorial diameter (*P*/*E*) ratio oblate, outline straight-triangular to slightly concave-triangular in polar view, bean-shaped in equatorial view (convex distal face versus concave proximal face); equatorial diameter 32–38 µm in LM, 27–34 µm in SEM, polar axis 9–15 µm in LM; triporate, pori positioned sub-apically on the distal polar face, pori elliptic, 4.5–6.0 µm in diameter, pori equipped with opercula; exine 1.7–2.5 µm thick in LM, nexine thinner than sexine; pollen wall semitectate; sculpture reticulate in LM, reticulate to perforate in SEM; distal face reticulate with broad muri and elliptic to triangular to polygonal lumina, 18–25 lumina per 100 µm^2^ at central distal face, 0–6 nanogemmae free-standing columellae per lumina (SEM); proximal face reticulate to perforate, lumina/perforations elliptic to triangular to polygonal, 0–6 nanogemmae free-standing columellae per lumina; central polar areas and interapertural areas reticulate, sculpture becoming microreticulate to perforate towards apices; opercula with nanoverrucate to granulate sublayer and distinct microreticulate supra-layer (SEM).

#### Remarks

The first LM and SEM micrographs showing pollen of this taxon are by Harley and Hall (1991, plate 4, figures 32 [SEM] and 33 [LM]). The same SEM micrograph is shown in Harley (1999, plate 1, figure 12), and again along with two additional LM micrographs and two attached grains under SEM in Harley (1996, plate 16, figures C and F [SEM], and G and H [LM]). These are all repeated in Harley and Baker (2001, figures 77 and 82 [SEM], 80 and 81 [LM]). A new SEM detail is provided in Harley and Dransfield (2003, figure 11). In total, five or six grains were illustrated using either LM or SEM micrographs, but no TEM micrograph has been presented thus far. All the pollen grains of this taxon illustrated by Harley (1996), Harley and Baker (2001) and Harley and Dransfield (2003) originate from the same herbarium sample (Tuley, s.n. [K]). All other previously published micrographs showing alleged pollen of *Sclerosperma mannii* originated from misidentified specimens (see Table IV). The LM and SEM based pollen morphology of the Tuley s.n. (K) sample is similar to that now observed in the van der Burgt 1958 (K) sample. The only difference is that the free-standing columellae are more prominent and more frequent in the specimen collected by Tuley versus that collected by van der Burgt, a feature comparable to what is observed in the *S*. *walkeri* pollen from the Leonard 1614 (BR) sample (compare figure 11 in Harley and Dransfield [2003], with Figures 2E and 6E this study).

**Table IV.** Affiliation of previously illustrated *Sclerosperma* pollen.

Taxon (Type)	Sample (herbarium)	Figured in	Noted as	Micrograph
*Sclerosperma mannii*	Tuley, s.n. (K)	Harley and Dransfield (2003)	*Sclerosperma mannii*	Figure 11 (SEM)
	Tuley, s.n. (K)	Harley and Baker (2001)	*Sclerosperma mannii*	Figures 77 and 82 (SEM), 80 and 81 (LM)
	Tuley, s.n. (K)	Harley (1999)	*Sclerosperma mannii*	Plate 1, figure 12 (SEM)
	Tuley, s.n. (K)	Harley (1996)	*Sclerosperma mannii*	Plate 16, figures C and F (SEM), G and H (LM)
	Tuley, s.n. (K)	Harley and Hall (1991)	*Sclerosperma mannii*	Plate 4, figures 32 (SEM), 33 (LM)
*Sclerosperma profizianum* (Type A)	Gillett, 279a (K)	Dransfield et al. (2008)	*Sclerosperma mannii*	Page 391, figure b (SEM)
	Gillett, 279a (K)	Harley (2004)	*Sclerosperma mannii*	Figure 6F (SEM)
	Gillett, 279a (K)	Harley and Dransfield (2003)	*Sclerosperma mannii*	Figures 10, 16 and 17 (SEM), 13 and 14 (LM), 15 and 19 (TEM)
*Sclerosperma profizianum* (Type B)	Profizi, 841 (K)	Dransfield et al. (2008)	*Sclerosperma profizianum*	Page 391, figures a (SEM), c and d (LM)
	Profizi, 841 (K)	Harley (2004)	*Sclerosperma gilletii*	Figure 6C (SEM), 6D and 6E (LM)
	Profizi, 841 (K)	Harley and Dransfield (2003)	*Sclerosperma gilletii*	Figure 12 (SEM)
*Sclerosperma profizianum* (Type C)	Hall & Enti, GC36150 (K)	Harley and Dransfield (2003)	*Sclerosperma mannii*	Figure 18 (TEM)
	Hall & Enti, GC36150 (K)	Harley and Baker (2001)	*Sclerosperma mannii*	Figures 78 and 79 (SEM)
	Hall & Enti, GC36150 (K)	Harley (1996)	*Sclerosperma mannii*	Plate 16, figures D and E (SEM)
*Sclerosperma walkeri*	Leonard, 1614 (?)	Sowunmi (1972)	*Sclerosperma mannii*	Plate 3, figure 8 (LM); plate 4, figure 1 (LM)

Note: Species affiliation according to Van Valkenburg et al. (2008). The drawings presented by Erdtman and Singh (1957) are not included in this table since they are not affiliated to any of the three *Sclerosperma* samples used in their study.

**Figure 3.**
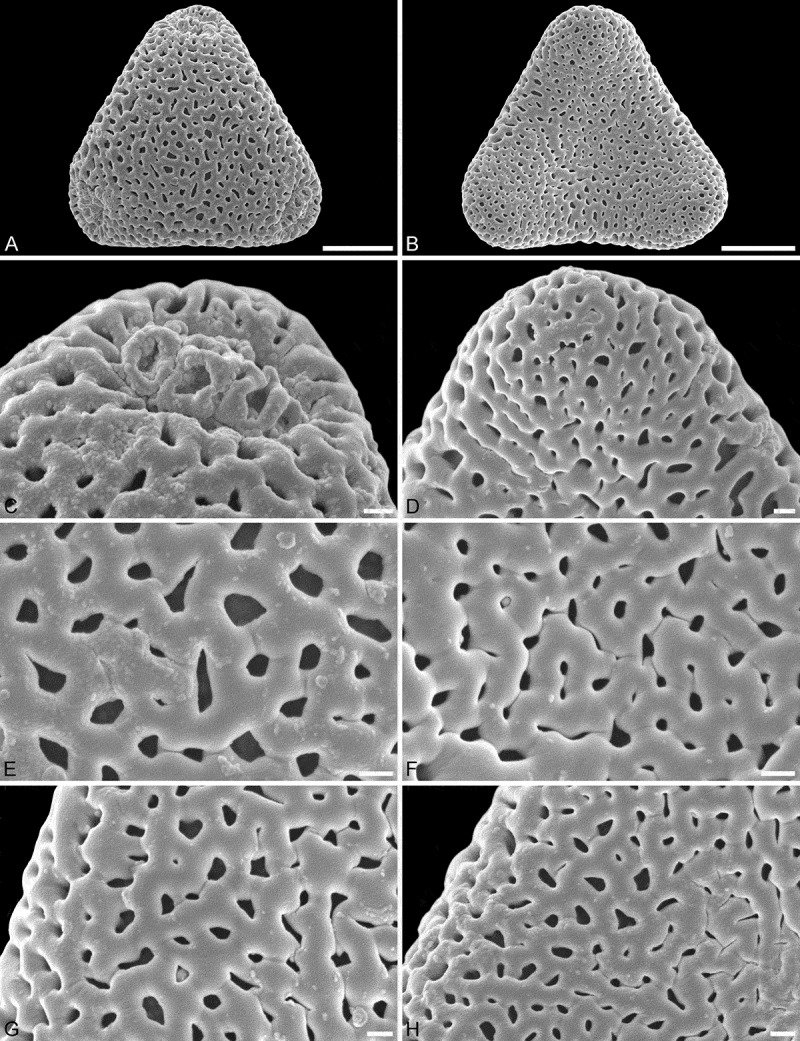
SEM micrographs of *Sclerosperma* *profizianum*, Type A (from DR Congo, coll. Gillet, 279a [WAG]). **A, C, E, G.** Pollen in polar view, distal side. **B, D, F, H.** Pollen in polar view, proximal side. **C, D.** Close-ups of apices (aperture on distal side). **E, F.** Close-ups of central polar areas (reticulum narrower on proximal side). **G, H.** Close-ups of interapertural areas. Scale bars – 10 µm (A–B), 1 µm (C–H).

**Figure 4.**
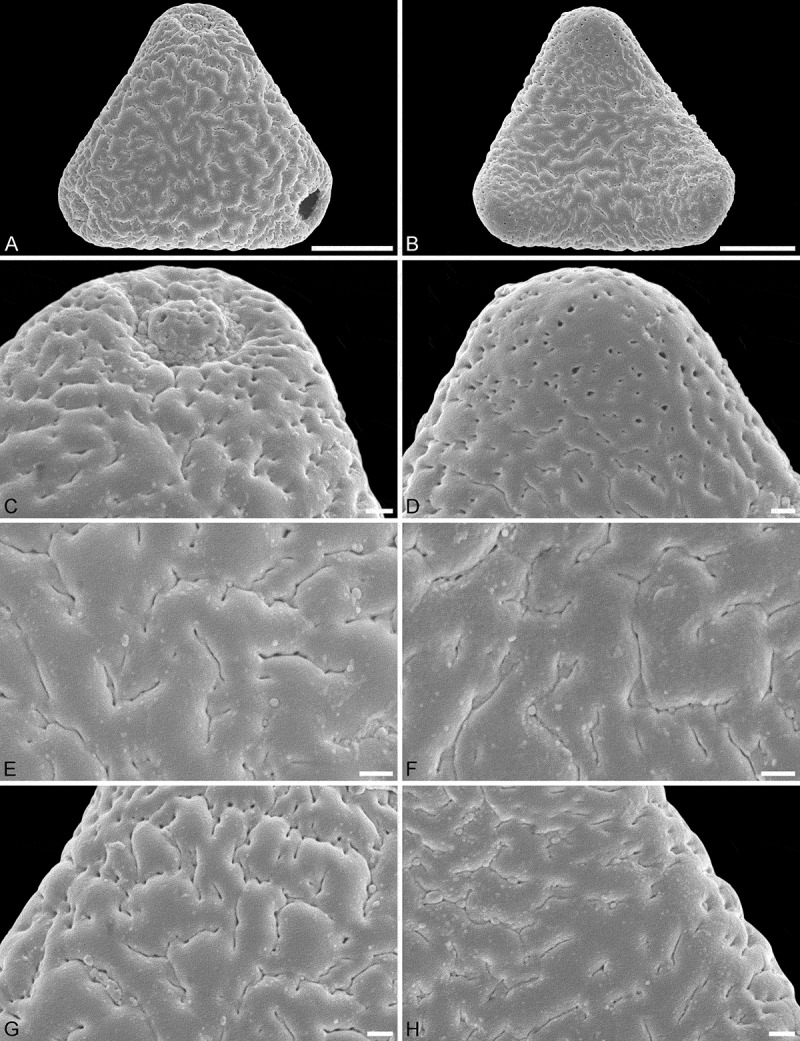
SEM micrographs of *Sclerosperma* *profizianum*, Type B (from Angola, coll. Grobbelaar, s.n. [K]). **A, C, E, G.** Pollen in polar view, distal side. **B, D, F, H.** Pollen in polar view, proximal side. **C, D.** Close-ups of apices (aperture on distal side). **E, F.** Close-ups of central polar areas. **G, H.** Close-ups of interapertural areas. Scale bars – 10 µm (A–B), 1 µm (C–H).

**Figure 5.**
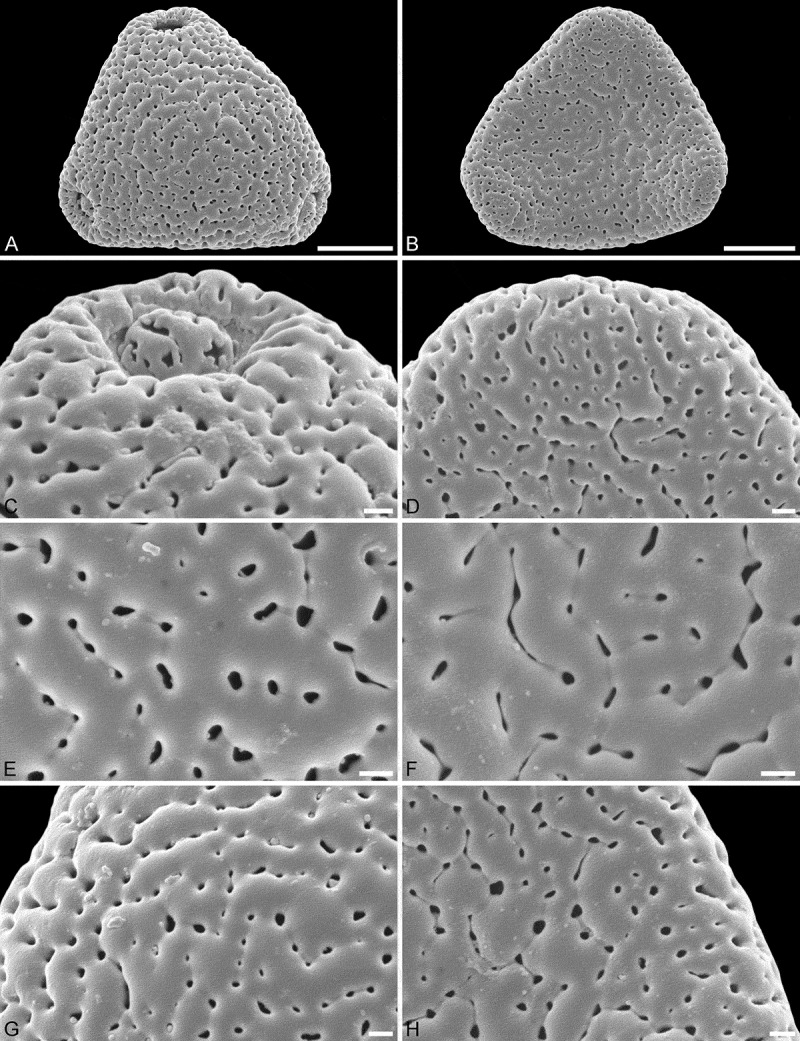
SEM micrographs of *Sclerosperma profizianum*, Type C (from Ghana, coll. Hall & Enti, GC 36150 [K], [**A, C, E, G**]; from R Congo, coll. Profizi, 841c2 [K], [**B, D, F, H**]). **A, C, E, G.** Pollen in polar view, distal side. **B, D, F, H.** Pollen in polar view, proximal side. **C, D.** Close-ups of apices (aperture on distal side). **E, F.** Close-ups of central polar areas. **G, H.** Close-ups of interapertural areas. Scale bars – 10 µm (A–B), 1 µm (C–H).

**Figure 6.**
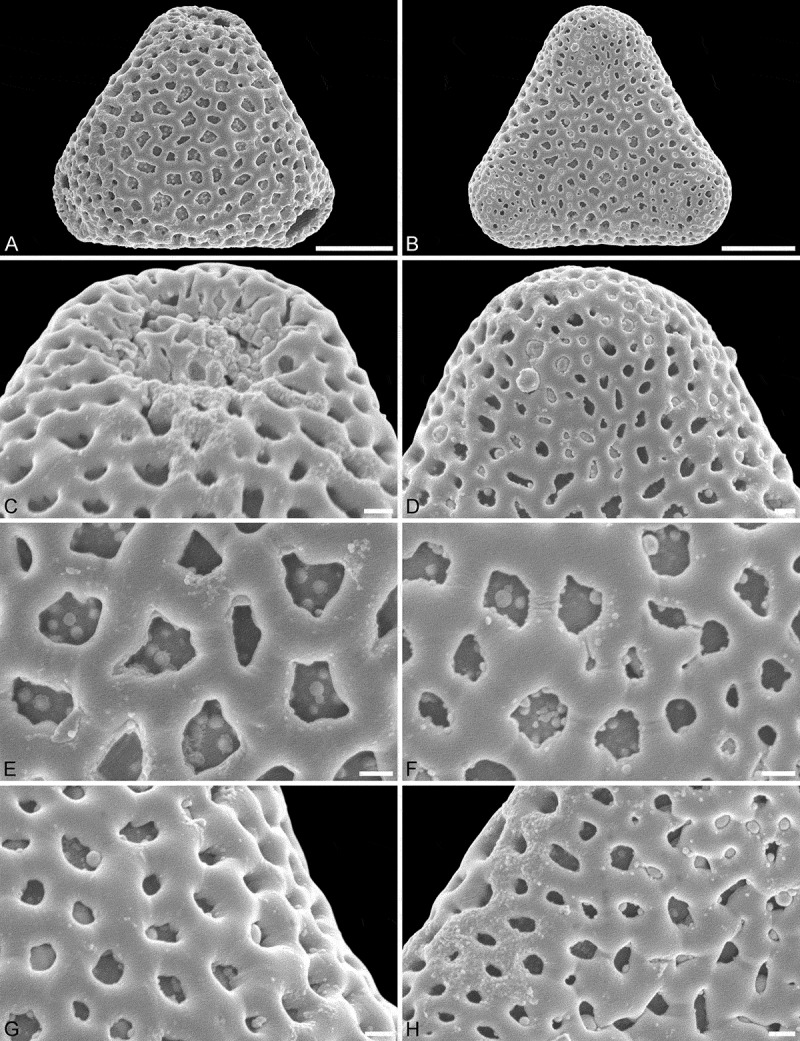
SEM micrographs of *Sclerosperma walkeri* (from DR Congo, coll. Leonard, 1614 [BR]). **A, C, E, G.** Pollen in polar view, distal side. **B, D, F, H.** Pollen in polar view, proximal side. **C, D.** Close-ups of apices (aperture on distal side). **E, F.** Close-ups of central polar areas (reticulum narrower on proximal side). **G, H.** Close-ups of interapertural areas. Scale bars – 10 µm (A–B), 1 µm (C–H).

*Species* Sclerosperma profizianum *Valk.* *et Sunderl.*

(Figures 1F–Y, 3–5; Table III)

#### Note

We encountered three pollen morphologies when analysing pollen grains from different herbarium specimens assigned to this taxon. The pollen morphologies are here described individually as Type A, B and C.

*Type A (Gillet, 279a [WAG]; holotype)*

(Figures 1F–J, 3; Table III)

#### Description

Pollen, monad, heteropolar, *P*/*E* ratio oblate, outline straight-triangular to slightly concave-triangular in polar view, bean-shaped in equatorial view (convex distal face versus concave proximal face); equatorial diameter 35–40 µm in LM, 29–35 µm in SEM, polar axis 10–14 µm in LM; triporate, pori positioned sub-apically on the distal polar face, pori elliptic, 5.0–8.5 µm in diameter, pori equipped with opercula; exine 1.7–2.5 µm thick in LM, 2.1–2.5 µm thick in SEM, nexine thinner than sexine, nexine 0.5–0.8 µm thick in SEM, sexine 1.2–1.8 µm thick in SEM; pollen wall semitectate; sculpture reticulate in LM, microreticulate to perforate in SEM; distal face microreticulate with broad muri and elliptic to triangular to polygonal lumina, 30–35 lumina per 100 µm^2^ at central distal face (SEM); proximal face microreticulate to perforate, lumina/perforations elliptic to circular or slit-like; central polar areas and interapertural areas microreticulate, sculpture becoming nanoreticulate to perforate towards apices; opercula with nanoverrucate to granulate sublayer and a distinct reticulate supra-layer (SEM).

#### Remarks

The first LM, SEM and transmission electron microscopy (TEM) micrographs showing pollen of this taxon are by Harley from the isotype (Gillet, 279a [K]) of the Kew herbarium (Harley & Dransfield 2003; figures 10, 16 and 17 [SEM], 13 and 14 [LM], and 19 [TEM]). The same SEM micrograph is shown in Harley (2004, figure 6F), and an additional SEM micrograph is provided in Dransfield et al. (2008, p. 391, figure b). All previously illustrated pollen grains that are here referred to *S. profizianum* (pollen Type A) were formerly assigned to *S. mannii* (see Table IV).

*Type B (Grobbelaar, s.n. [K])*

(Figures 1K–O, 4; Table III)

#### Description

Pollen, monad, heteropolar, *P*/*E* ratio oblate, outline straight-triangular to slightly concave-triangular in polar view, bean-shaped in equatorial view (convex distal face versus concave proximal face); equatorial diameter 32–38 µm in LM, 30–34 µm in SEM, polar axis 10–12.5 µm in LM; triporate, pori positioned sub-apically on the distal polar face, pori circular to elliptic, 4.0–5.5 µm in diameter, pori equipped with opercula; exine 1.7–2.5 µm thick in LM, nexine thinner than sexine; pollen wall tectate; sculpture rugulate in LM, fossulate, rugulate/verrucate and perforate in SEM; distal face fossulate with tiny circular to slit-like perforations aligned within the fossulae, sinuous fossulae outlining irregular shaped rugulae/verrucae (SEM); proximal face fossulate with tiny circular to slit-like perforations aligned within the fossulae, sinuous fossulae outlining irregular shaped rugulae/verrucae, sculpture becoming microrugulate to nanorugulate/verrucate and perforate towards apices; opercula with nanoverrucate to granulate sublayer and perforate supra-layer (SEM).

#### Remarks

Pollen of this type was originally noted as *Sclerosperma gilletii* by Harley and Dransfield (2003, figure 12 [SEM]) and Harley (2004, figures 6C [SEM], 6D and 6E [LM]). The same micrographs were provided in Dransfield et al. (2008, p. 391, figures a [SEM], c and d [LM]), but then affiliated to *S. profizianum* following description of that new species in the publication of Van Valkenburg et al. (2008). It is interesting that our study of the same herbarium material (Profizi, 841 [K]), but different flower, did not give the same pollen Type B as that figured by Harley and Dransfield (2003), Harley (2004) and Dransfield et al. (2008), but pollen Type C (Table IV). This suggests that *S. profizianum* Type B pollen and *S. profizianum* Type C pollen are not just very close in morphology, but might be produced by the same individual plant, or there is some sort of sampling error.

*Type C (Hall & Enti, GC36150 [K]; Profizi, 841 [K])*

(Figures 1P–Y, 5; Table III)

#### Description

Pollen, monad, heteropolar, *P*/*E* ratio oblate, outline straight-triangular to slightly convex-triangular in polar view, bean-shaped in equatorial view (convex distal face versus concave proximal face); equatorial diameter 37–42 µm in LM, 31–39 µm in SEM, polar axis 10–16 µm in LM; triporate, pori positioned sub-apically on the distal polar face, pori circular to elliptic, 4.0–6.5 µm in diameter, pori equipped with opercula; exine 1.7–2.5 µm thick in LM, nexine thinner than sexine; pollen wall tectate; sculpture scabrate in LM, perforate, rugulate/verrucate and fossulate in SEM; distal face perforate, perforations elliptic to slit-like, perforations often aligned in sinuous rows, rows of perforations outlining irregular shaped rugulae/verrucae (SEM); proximal face perforate and fossulate, perforations elliptic to slit-like, perforations often aligned in sinuous rows, rows of perforations and fossulae outlining irregular shaped rugulae/verrucae, sculpture becoming microrugulate to nanorugulate/verrucate and perforate towards apices; opercula with nanoverrucate to granulate sublayer and perforate supra-layer (SEM).

#### Remarks

The first SEM micrographs showing pollen of this taxon are by Harley (1996, pl. 16, figures D and E). The same two SEM micrograph are repeated in Harley and Baker (2001, figures 78 and 79). A single TEM showing the aperture region and operculum is presented in Harley and Dransfield (2003, figure 18). All previously illustrated pollen grains belonging to this taxon (pollen Type C) were formerly assigned to *Sclerosperma mannii* (see Table IV).

*Species* Sclerosperma walkeri *A. Chev. (Leonard, 1614 [BR])*

(Figures 1D', 6; Table III)

#### Description

Pollen, monad, heteropolar, *P*/*E* ratio oblate, outline straight-triangular to slightly concave-triangular in polar view, bean-shaped in equatorial view (convex distal face versus concave proximal face); equatorial diameter 35–40 µm in LM, 30–35 µm in SEM, polar axis 15–19 µm in LM; triporate, pori positioned sub-apically on the distal polar face, pori elliptic, 5.0–8.0 µm in diameter, pori equipped with opercula; exine 1.7–2.5 µm thick in LM, nexine thinner than sexine; pollen wall semitectate; sculpture reticulate in LM, reticulate to perforate in SEM; distal face reticulate with broad muri and elliptic to triangular to polygonal lumina, 16–25 lumina per 100 µm^2^ at central distal face, 0–6 nanogemmae free-standing columellae per lumina (SEM); proximal face reticulate to perforate, lumina/perforations elliptic to triangular to polygonal, 0–6 nanogemmae free-standing columellae per lumina; central polar areas and interapertural areas reticulate, sculpture becoming microreticulate to perforate towards apices; opercula with nanoverrucate to granulate sublayer and distinct microreticulate supra-layer (SEM).

#### Remarks

Two LM micrographs showing pollen of this taxon are provided by Sowunmi (1972, plate 3, figure 8 and plate 4, figure 1), but assigned to *S*. *mannii* (Table IV).

## Discussion

### Differentiating *Sclerosperma pollen*

Based on the pollen morphology of *Sclerosperma* presented herein it is clear that there are at least four different pollen morphologies produced by the three extant taxa. *Sclerosperma mannii* and *S. walkeri* share similar pollen morphology, and are difficult to distinguish from each other in both LM and SEM. *Sclerosperma profizianum* produced three different pollen morphologies (Types A, B, and C), distinguishable from each other and from *S. mannii* and *S. walkeri*, particularly in SEM.

Using LM only, *Sclerosperma* pollen can be divided into reticulate (including *S. manni, S. profizianum* Type A, and *S. walkeri*) and non-reticulate (including *S. profizianum* Type B and C; Table III). The reticulate pollen are further divided into coarsely reticulate (including *S. mannii* and *S. walkeri*) versus finely reticulate (*S. profizianum* Type A; compare Figure 1A and 1Z with Figure 1F). Our measurements indicate the coarsely reticulate pollen of *S. mannii* and *S. walkeri* can be set apart using the length of their polar axis, which is longer in the pollen of *S. walkeri* (15–19 µm) than in *S. mannii* (9–15 µm). The non-reticulate *Sclerosperma* pollen grains are distinguishable by having rugulate (*S.* *profizianum* Type B) or scabrate (*S. profizianum* Type C) sculpture. The rugulate *S. profizianum* Type B pollen is usually smaller than that of *S. profizianum* Type C (Table III), and the outline of the pollen in polar view is more convex-triangular in *S. profizianum* Type C versus concave-triangular in *S. profizianum* Type B.

Applying additional SEM, there are a number of details separating the three different *Sclerosperma* *profizianum* pollen types from each other and from the pollen of *S. mannii* and *S. walkeri*. The magnification obtained using SEM shows that *S. mannii* and *S. walkeri* pollen is more or less identical (Table III). The only noticeable sculpture difference, so far, is that the free-standing columellae in *S. walkeri* pollen are more frequent and conspicuous than in *S. mannii* pollen (compare Figure 2E and 2F with Figure 6E and 6F). Still, the *S*. *mannii* and *S. walkeri* pollen are easily distinguished from the three *S. profizianum* pollen types. The *S. mannii* and *S. walkeri* pollen is reticulate with 16–25 lumina per 100 µm^2^ at the central distal face versus microreticulate with 30–35 lumina per 100 µm^2^ in *S. profizianum* Type A (compare Figures 2E and 6E with Figure 3E). The *S. profizianum* Type C pollen is perforate and rugulate/verrucate with 45–55 perforations per 100 µm^2^ at the central polar face, and the *S. profizianum* Type B pollen is fossulate and rugulate/verrucate with the perforations hidden in the fossulae (compare Figure 5E with Figure 4E; Table III). Furthermore, the *S. profizianum* Type A pollen has regularly distributed elliptic to circular or slit-like lumina versus perforations aligned in sinuous rows in *S. profizianum* Type C versus tiny perforations aligned (hidden) within the fossulae in *S. profizianum* Type B pollen. Also, the operculum in *S. profizianum* Type A pollen has a clear reticulate supra-layer versus perforate in both *S. profizianum* Types B and C pollen (compare Figure 3C with Figures 4C and 5C).

### Pollen morphology and taxonomic resolution

The ‘identical’ pollen of *Sclerosperma mannii* and *S. walkeri* are from sites near the centre of distribution for the genus (see map 1 in Van Valkenburg et al. 2008). Pollen of *S. profizianum* Type C is from a disjunct population in Ghana and is also found in a more centrally located population in Republic of the Congo. The two other *S. profizianum* pollen Types, A and B, are from the southern edge of the distribution of that species/genus near the border between Democratic Republic of the Congo and Angola.

There are many examples of clades in which species can be clearly separated on the basis of plant body and reproductive parts, but the pollen produced by them are similar or identical to each other morphologically (termed stenopalynous taxa, see Halbritter et al. 2018). However, it is not common for a single species to produce two or more distinct pollen morphologies (in sculpture and/or size), unless the plants are heterostylous (for a list of such genera see table 1 in Ganders 1979). There are no hints in the literature that heterostyly occurs in *Sclerosperma*.

We did not discover more than a single pollen type from a particular or several anthers out of an individual *Sclerosperma* flower; the pollen morphology observed within an anther or anthers were distinct and consistent within an individual or between flowers from the same herbarium sample. The pollen morphology of *S. profizianum* Types B and C suggests that they are very close, and based on previous work by Harley on some of the same herbarium material [Profizi, 841 (K)] it is even possible that they were produced by the same plant. Still, M.M. Harley only figured a single pollen grain in SEM (Harley & Dransfield 2003; Harley 2004; Dransfield et al. 2008) and therefore a sampling error or contamination cannot be excluded. The material studied might also have been assigned to the wrong collector information. Disregarding all that, and assuming that the Types B and C pollen originate from the same plant or taxon it is clear that *S. *profizianum** still seems to produce two undoubtedly different pollen types: (1) the microreticulate pollen Type A and (2) the fossulate/perforate Type B/C pollen.

The reason *Sclerosperma* *profizianum* seems to produce different pollen types is unclear currently, but we can think of two possible explanations for this situation. First, *S. profizianum* is a unique taxon that produces different pollen types without being heterostylous. This seems very unlikely, but cannot be excluded. Second, the currently accepted species definitions in *Sclerosperma* do not reflect its actual biological diversity. *Sclerosperma* *profizianum* may be composed of more than a single natural species, or at least it may be in the process of genetic diversification related to its disjunct distribution and marginal occurrences. This could explain observed variations in pollen morphology, including the intermediate sculpture features of *S. profizianum* pollen Type A (microreticulate versus reticulate in *S. mannii*/*S. walkeri*, and perforate in *S. profizianum* Type B/C). Whatever the explanation, it cannot be resolved from the data presented here.

## Conclusion and outlook

Combined LM and SEM analyses demonstrate that there are four different pollen morphologies produced by *Sclerosperma. Sclerosperma mannii* and *S. walkeri* share similar pollen morphologies, but S. *profizianum* produces three different pollen types. Despite the detailed pollen work presented here and all the available literature regarding Arecaceae taxonomy, pollen morphology and phylogeny (e.g. Dransfield et al. 2008), there is still much that needs to be studied. For *Sclerosperma*, it would be vital to explore intrageneric relationships and conduct a molecular phylogenetic study using several representatives from each alleged species to see how they align in a phylogenetic tree. In this sense it would be interesting to see if the *S. mannii* samples group together and appear as sister taxon to the *S. walkeri* samples, and if all the *S*. *profizianum* fall into one branch or are not clearly resolved suggesting some sort of species differentiation. When conducting such a study it would be highly informative to analyse pollen morphology from the same plants used for the molecular phylogeny and plot the pollen on the resulting tree. In such a case the evolution of pollen morphology in *Sclerosperma* could be resolved. Also, the comprehensive LM and SEM based pollen morphology presented here will now allow for a better determination of fossil *Sclerosperma* pollen grains and the re-analyses of fossil material previously affiliated to this genus.

## References

[CIT0001] APG IV 2016 An update of the Angiosperm Phylogeny Group classification for the orders and families of flowering plants: APG IV. Botanical Journal of the Linnean Society 181: 1–20 doi:10.1111/boj.2016.181.issue-1.

[CIT0002] Bourobou Bourobou PH, Niangadouma R, Issembe Y, Couvreur TLP. 2016 Two new records of palm species for Gabon: Sclerosperma profizianum Valk. & Sunder. and Eremospatha quiquecostulata Becc. Biodiversity Data Journal 4: e10187. doi:10.3897/BDJ.4e10187. PMC5139133 27956852

[CIT0003] Dransfield J, Uhl NW, Asmussen CB, Baker WJ, Harley MM, Lewis CE. 2008 Genera Palmarum. The evolution and classification of palms. Kew: Kew Publishing.

[CIT0004] Erdtman G, Sing G 1957 On the pollen morphology in *Sclerosperma mannii* . Bulletin du Jardin botanique de l’État a Bruxelles 27: 217–220. doi:10.2307/3666958.

[CIT0005] Ganders FR 1979 The biology of heterostyly. New Zealand Journal of Botany 17: 607–635. doi:10.1080/0028825X.1979.10432574.

[CIT0006] Grímsson F, Grimm GW, Zetter R 2017 Tiny pollen grains: First evidence of Saururaceae from the Late Cretaceous of western North America. PeerJ 5: e3434. doi:10.7717/peerj.3434. 28626610 PMC5472062

[CIT0007] Grímsson F, Grimm GW, Zetter R 2018 Evolution of pollen morphology in Loranthaceae. Grana 57: 16–116. doi:10.1080/00173134.2016.1261939. 29386990 PMC5771552

[CIT0008] Halbritter H, Ulrich S, Grímsson F, Weber M, Zetter R, Hesse M, Buchner R, Svojtka M, Frosch-Radivo A 2018 Illustrated pollen terminology. Second edition Vienna: Springer.

[CIT0009] Harley MM 1996 Palm pollen and the fossil record. PhD Thesis, University of East London, London, UK.

[CIT0010] Harley MM 1999 Tetrad variation: Its influence on pollen form and systematics in the Palmae In: Kurmann MH, Hemsley AR, eds. Evolution of plants architecture, 289–304. Kew: Kew Publishing.

[CIT0011] Harley MM 2004 Triaperturate pollen in the monocotyledons: Configurations and conjectures. Plant Systematics and Evolution 247: 75–122. doi:10.1007/s00606-003-0107-x.

[CIT0012] Harley MM, Baker WJ 2001 Pollen aperture morphology in Arecaceae: Application within phylogenetic analyses, and a summary of the fossil record of palm-like pollen. Grana 40: 45–77. doi:10.1080/00173130152591877.

[CIT0013] Harley MM, Dransfield J 2003 Triporate pollen in the Arecaceae. Grana 42: 3–19. doi:10.1080/00173130310008535.

[CIT0014] Harley MM, Hall DH 1991 Pollen morphology of the African palms. Palaeoecology of Africa and the surrounding islands 22: 11–25.

[CIT0015] Punt W, Hoen PP, Blackmore S, Nilsson S, Le Thomas A 2007 Glossary of pollen and spore terminology. Review of Palaeobotany and Palynology 143: 1–81. doi:10.1016/j.revpalbo.2006.06.008.

[CIT0016] Sowunmi MA 1972 Pollen morphology of the Palmae and its bearing on taxonomy. Review of Palaeobotany and Palynology 13: 1–80. doi:10.1016/0034-6667(72)90044-9.

[CIT0017] Van Valkenburg JLCH, Sunderland TCH, Couvreur TLP 2008 A revision of the genus *Sclerosperma* (Arecaceae). Kew Bulletin 63: 75–86. doi:10.1007/s12225-007-9002-x.

[CIT0018] WCSP 2018 World Checklist of Selected Plant Families. Facilitated by the Royal Botanic Gardens, Kew. Published on the Internet, http://wcsp.science.kew.org/; accessed 6 February 2018.

